# Conductive-probe atomic force microscopy characterization of silicon nanowire

**DOI:** 10.1186/1556-276X-6-110

**Published:** 2011-01-31

**Authors:** José Alvarez, Irène Ngo, Marie-Estelle Gueunier-Farret, Jean-Paul Kleider, Linwei Yu, Pere Rocai Cabarrocas, Simon Perraud, Emmanuelle Rouvière, Caroline Celle, Céline Mouchet, Jean-Pierre Simonato

**Affiliations:** 1 Laboratoire de Génie Electrique de Paris, CNRS UMR 8507, SUPELEC, Univ P-Sud, UPMC Univ Paris 6, 11 rue Joliot-Curie, Plateau de Moulon, 91192 Gif-sur-Yvette Cedex, France; 2 Laboratoire de Physique des Interfaces et des Couches Minces, Ecole Polytechnique, CNRS, 91128 Palaiseau, France; 3 CEA, Laboratoire des Composants pour la Récupération d'Energie (LITEN), 17 rue des Martyrs, 38054 Grenoble Cedex 9, France

## Abstract

The electrical conduction properties of lateral and vertical silicon nanowires (SiNWs) were investigated using a conductive-probe atomic force microscopy (AFM). Horizontal SiNWs, which were synthesized by the in-plane solid-liquid-solid technique, are randomly deployed into an undoped hydrogenated amorphous silicon layer. Local current mapping shows that the wires have internal microstructures. The local current-voltage measurements on these horizontal wires reveal a power law behavior indicating several transport regimes based on space-charge limited conduction which can be assisted by traps in the high-bias regime (> 1 V). Vertical phosphorus-doped SiNWs were grown by chemical vapor deposition using a gold catalyst-driving vapor-liquid-solid process on higly *n*-type silicon substrates. The effect of phosphorus doping on the local contact resistance between the AFM tip and the SiNW was put in evidence, and the SiNWs resistivity was estimated.

## Introduction

Silicon nanowires (SiNWs) are promising nanostructures which are expected to be integrated in building blocks for future microelectronics and optoelectronics devices [1-3]. Indeed, multiple studies have already shown the great potential of SiNWs as functional element to develop transistors [4], biosensors [5], memory applications [6], and as electrical interconnects [7]. In addition, SiNWs offer an interesting geometry for light trapping and carrier collection which gives place to intensive investigations in the photovoltaic field [8,9].

Several approaches and strategies exist to grow, deploy, and assemble SiNWs [10,11]. In order to guide them, and more specifically to control the electrical properties of SiNWs, it is required to characterize their electronic transport properties.

Conductive-probe atomic force microscopy (CP-AFM) [12] reveals itself as a powerful current sensing technique for electrical characterizations in small-scale technologies, which could help us to explore the electrical properties and to reveal local conductivity fluctuations in SiNWs.

In this study, the authors focus on the CP-AFM characterization of horizontal SiNWs produced via in-plane solid-liquid-solid (IPSLS) method and phosphorus-doped vertical SiNWs obtained through vapor-liquid-solid (VLS) technique. Local resistance mapping and local current-voltage (*I*-*V*) measurements have been performed to evaluate the electrical properties of such semiconducting SiNWs.

## Experimental details

### Silicon nanowires

#### Horizontal SiNWs

**Figure 1:**
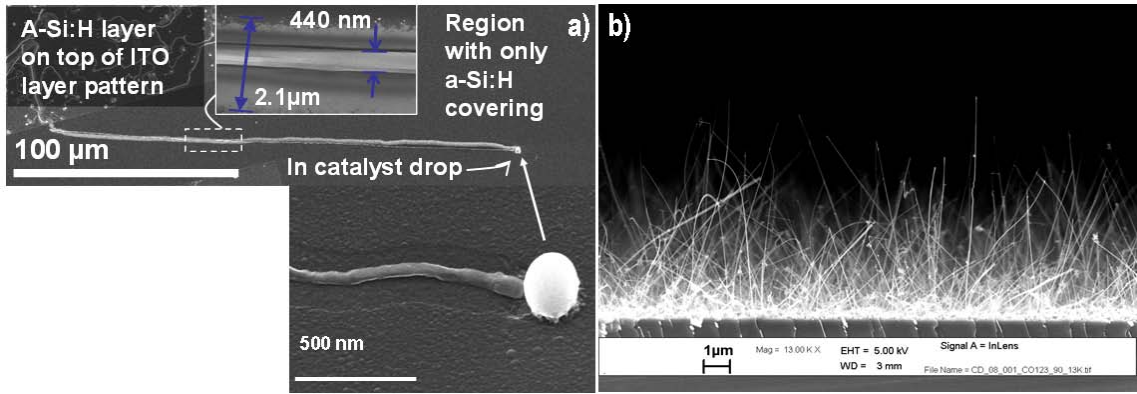
**SEM picture illustrating(a) **a single horizontal Si wire and **(b) **a carpet of vertical SiNWs.

The IPSLS [10,13,14] approach, using indium (In) catalyst droplets and a hydrogenated amorphous silicon (a-Si:H) layer, was used to grow horizontal SiNWs. More precisely, In catalyst droplets were prepared by superficial reduction of an indium tin oxide (ITO) layer, which was then coated by an a-Si:H layer. The growth activation of SiNWs is done during an annealing process at temperatures in the range of 300-500°C. The mechanism for obtaining horizontal SiNWs is guided by the liquid In drop which interacts with the predeposited a-Si:H transforming it into crystalline SiNWs. Figure 1a illustrates a scanning electron microscopy (SEM) image of a horizontal Si wire of 400-nm diameter which extends over one hundred of microns. The In catalyst is still visible at the end of the wire.

### Vertical SiNWs

*n*-Type phosphorous-doped SiNWs were grown by chemical vapor deposition through the gold-catalyzed VLS method as described in [15,16], on *n*-type silicon substrates (3-5 mΩ cm). The SiNW growth temperature was in the range of 500-650°C, and the *n*-type doping was achieved by adding PH_3 _to SiH_4_, with PH_3_/SiH_4 _ratios which can vary from 0 to 2 × 10^-2^. Subsequent to the growth, the catalyst was removed, and in some cases, a rapid thermal annealing at 750°C for 5 min was done to activate dopant impurities. SiNWs were then embedded into spin-on-glass matrix in order to be planarized by chemical-mechanical polishing [16].

Table 1 describes the samples that were electrically analyzed by CP-AFM. The samples were grown at the same temperature (500°C), and they differentiate themselves on the nominal doping concentration. Figure 1b illustrates a sample of vertical SiNWs on *n*-type Si wafer with diameters in the range of 50-100 nm. The length of wires after planarization was estimated around 1 μm.

**Table 1:** Sample description of vertical SiNWs analyzed by the CP-AFM technique

Sample name	Growth temp. (°C)	Description	Post-annealing treatment	Nominal impurity concentration
CD-08-001	500	Undoped SiNWs/*n*-type Si (100)	-	Undoped
CD-08-125	500	Doped SiNWs/*n*-type Si (100)	5 min at 750°C	[*P*] ≈ 1 × 10^18 ^cm^-3^
CD-08-021	500	Doped SiNWs/*n*-type Si (100)	5 min at 750°C	[*P*] ≈ 1 × 10^20 ^cm^-3^

### Conductive-probe atomic force microscopy

Local electrical measurements were performed using a Digital Instruments Nanoscope IIIa Multimode AFM associated with the home-made conducting probe extension called "Resiscope" [12]. This setup allows us to apply a stable DC bias voltage (from -10 to +10 V with 0.01 V resolution) to the device and to measure the resulting current flowing through the tip as the sample surface is scanned in contact mode. Local resistance values can be measured in the range of 10^2^-10^12 ^Ω, which allows investigations on a variety of materials [17,18] and devices [19,20]. Measurement accuracy based on calibrations is below 3% in the range of 10^2^-10^11 ^Ω, and it can reach 10% for higher resistance values.

Reliable and understandable electrical measurements through CP-AFM setup require a well-characterized conductive tip. Depending on the experimental conditions, the AFM conductive tip should be the most suitable in terms of serial resistance that must be taken into account in the electrical analysis of SiNWs. B-doped diamond- and PtIr-coated Si cantilevers, with an intermediate spring constant of about 2 N/m, prove to be suitable for our experimental conditions, since measured resistance values are mostly greater than their intrinsic resistances that are estimated at 5-10 and 0.3-1 kΩ, respectively.

**Figure 2:**
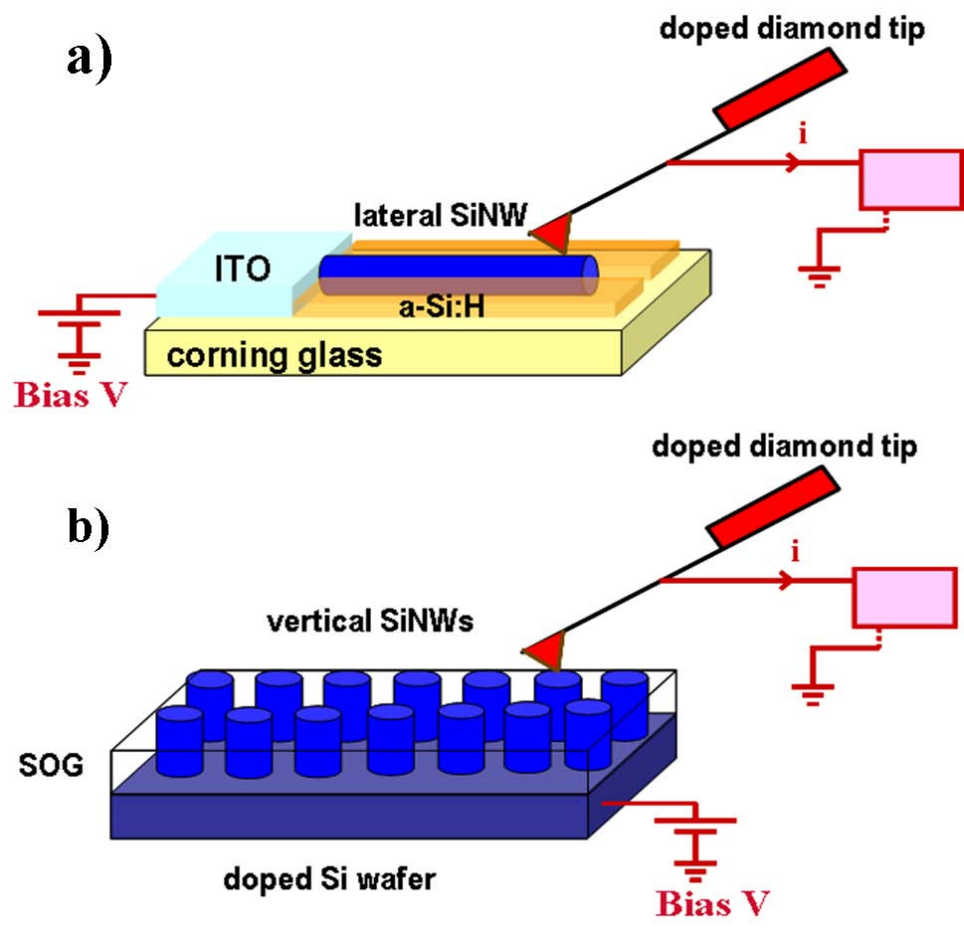
Sketch illustrating the details of CP-AFM measurements on (a) horizontal and (b) vertical SiNWs.

The CP-AFM details and more specifically the sample configuration and biasing are displayed in Figure 2. In case of horizontal SiNWs, the DC bias voltage was applied to the ITO pad, while for vertical SiNWs it was applied through the doped silicon wafer.

## Results and discussion

### Horizontal SiNWs

**Figure 3:**
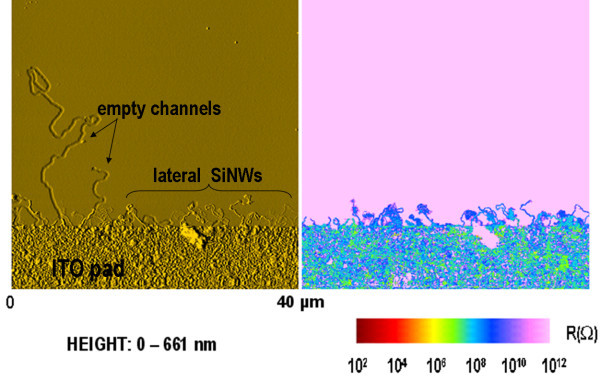
**40 × 40 μm^2 ^surface map illustrating the topography (left side) and the local resistance (right side) of horizontal SiNWs grown from In droplets obtained after reduction of ITO**.

Figure 3 shows a large AFM scan illustrating the topography and electrical image properties of the sample structure based on an ITO pad (bottom of the image) from the border of which in-plane nanowires are distinguishable. In addition, the topography allows it to point out long channels that were dug during the growth of SiNWs. Nevertheless, these long channels are empty and indeed they are not electrically discernable from the insulating a-Si:H layer that surrounds the wires. On the contrary, SiNWs show electrical conductivity when the wires are not broken or disconnected from the ITO pad.

**Figure 4:**
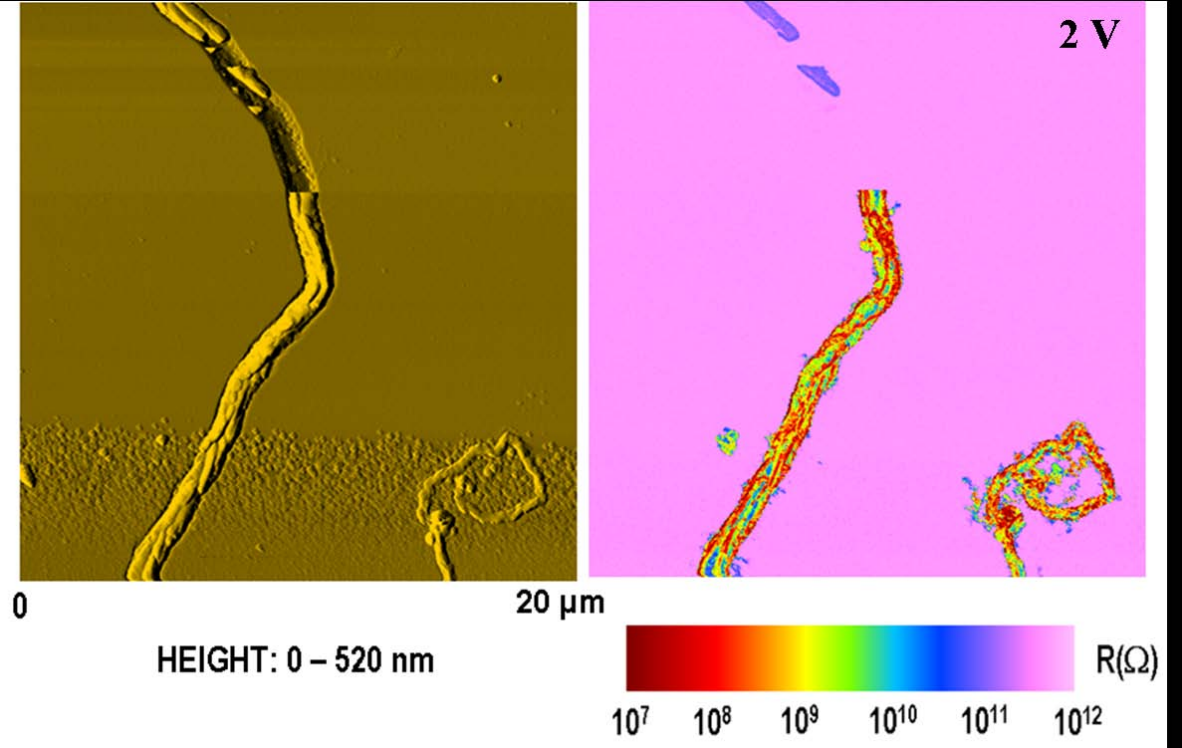
**Topography and local resistance maps illustrating a micrometer-wide horizontal silicon wire**. The electrical image was obtained under a bias of 2 V.

In Figure 4, a 20 × 20 μm^2 ^surface scan which displays the topography and the electrical properties of a micrometer-wide horizontal silicon oval shaped wire (1 μm wide and 300 nm thick) is presented. The topography points out an inhomogeneous surface morphology that is clearly confirmed by the local mapping of resistance. Indeed, conductive paths along the wire are put in evidence and linked to the topographic features of the wire envelope. The accuracy of these features depends essentially on convolution effects associated to the AFM tip shape. It seems reasonable that several SiNWs have been produced and have partially contributed to the growth of this long and wide silicon wire [10] explaining the electrical and surface microstructure.

In the same figure, the empty growth channel resulting from the unexpected cut of the wire with the AFM probe can also be noticed. Broken pieces of silicon remaining in the channel reveal a slight electrical conduction (10^11 ^Ω) although they are electrically isolated through the undoped a-Si:H layer (10^12 ^Ω). Possible explanations are that the whole surface of the remaining piece of silicon in contact with the a-Si:H layer fully contributes to decrease the electrical contact resistance or that the friction of the AFM tip induces charging effects which are electrically observable.

**Figure 5:**
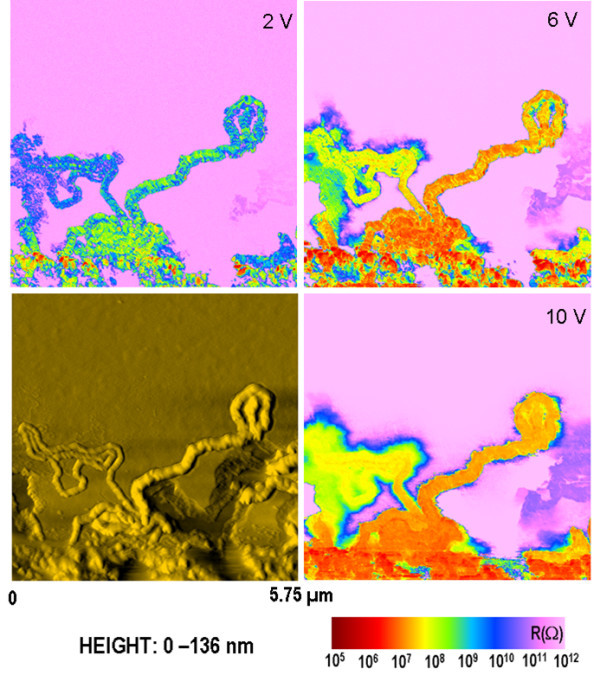
**Topography and local resistance maps depicting horizontal SiNWs randomly oriented**. The electrical measurements were done at different applied biases: 2, 6, and 10 V.

Horizontal SiNWs have also been characterized under different applied voltages. As illustrated in Figure 5, the local resistance maps were measured in the same region at 2, 6, and 10 V, respectively. The analysis of the electrical images points out a local resistance that decreases in function of the applied voltage. More specifically, the local resistance of SiNWs measured at 2 V decreases one order of magnitude at 6 V and two orders of magnitude at 10 V. Such behavior was also observed for negative applied biases. An interesting observation comes from the high bias regime (*V *> 2 V) which underlines the increase of local resistance of the wire versus its length. However, high bias regime can also broaden the electrical images of wires.

**Figure 6:**
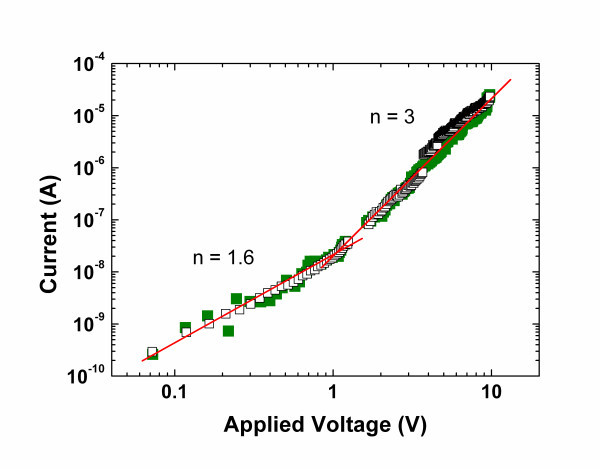
***I*-*V *measurement on individual SiNW measured by CP-AFM**.

In order to get more precise information about the variation of the local resistance in function of the applied bias, CP-AFM was locally used for investigating the *I*-*V *characteristics on individual SiNWs. Figure 6 displays a log-log plot of the *I*-*V *characteristics where two identifiable slopes are put in evidence. Indeed, the analysis of the slopes following a power-law dependence (*I *∝ *V*^*n*^) allows us to estimate two transport regimes with a transition around 1 V. The slope *n *= 1.6 (*V *< 1 V) points out charge injection which is a characteristic of a space-charge limited current (SCLC) [21]. The slope *n *= 3 (*V *> 1 V) indicates a trap-limited SCLC, that can be analyzed in the frame of a trap distribution with an increasing density of states toward the band edge. Interface and surface states in low-dimensional semiconductors such as nanowires are expected to be the most common defects, which greatly influence the electrical transport properties [22]. We also should keep in mind that SiNWs were here obtained thanks to an a-Si:H layer that is known to possess a quite large density of states in the gap, with exponential band tails.

### Vertical SiNWs

**Figure 7:**
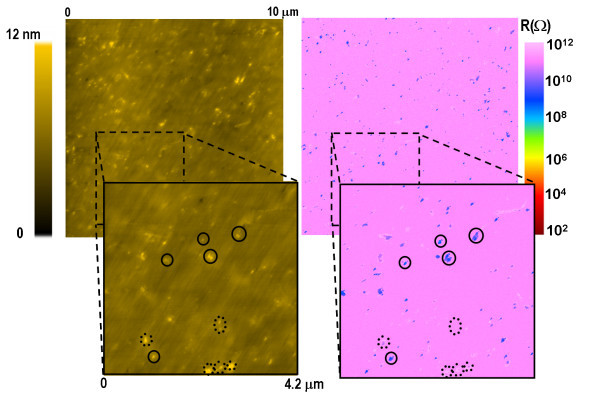
**Surface scan illustrating the topography (left) and the local resistance (right) performed on undoped vertical SiNWs (CD-08-001)**. Image zoom shows several examples of electrically conductive (full-line circle) and non-conductive (dot-line circle) SiNWs.

Figure 7 depicts a 10 × 10 μm^2 ^surface map that illustrates, from left to right, the topography and the electrical properties of undoped SiNWs (CD-08-001). The brightest spots (highest features) in the topography image represent the SiNWs which are generally well correlated with the conductive blue spots in the electrical image. However, the zoom (4.2 × 4.2 μm^2^) allows it to point out several examples of SiNWs which are not electrically conductive (dot-line circle) as distinct from those showing conductive properties (full-line circle). The oxide formation and the AFM tip loading force are possible reasons that could explain that SiNWs appear insulating in native.

**Figure 8:**
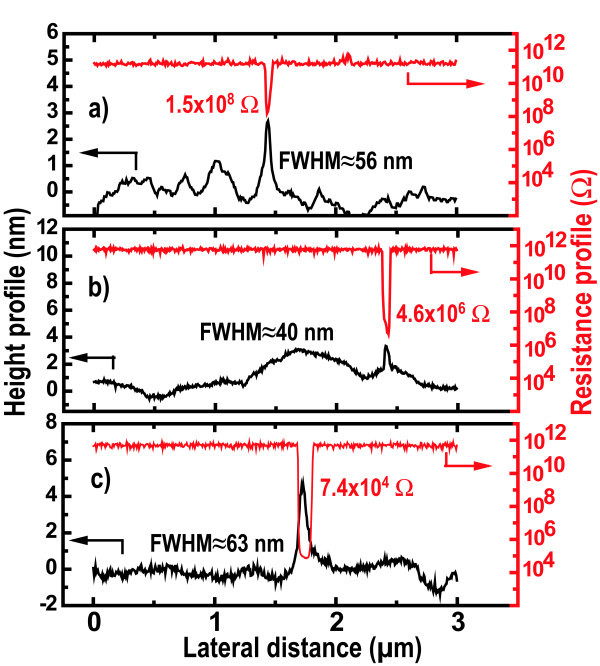
**Height and local resistance profile involving single SiNWs for different phosphorus doping levels **: **(a) **undoped, **(b) **[*P*] ≈ 1 × 10^18 ^cm^-3^, and **(c) **[*P*] ≈ 1 × 10^20 ^cm^-3^.

The three samples were carefully imaged, and a statistic was made in a few tenths of SiNWs. An example of cross-sectional profiles involving SiNWs is illustrated in Figure 8. The conducting wires are easily put in evidence with a decrease of the local resistance by several orders of magnitude with respect to the background signal. For the most highly doped sample, the local resistance of the SiNW drops by more than six orders of magnitude, whereas the intermediate doped and undoped samples show a decrease of four and three orders of magnitude, respectively. These measurements clearly point out that the SiNWs conductivity can be controlled by the incorporation of phosphorus impurities. However, the phosphorus doping efficiency and activation cannot be directly discussed through such measurements. Resistivity measurements are indeed required.

**Figure 9:**
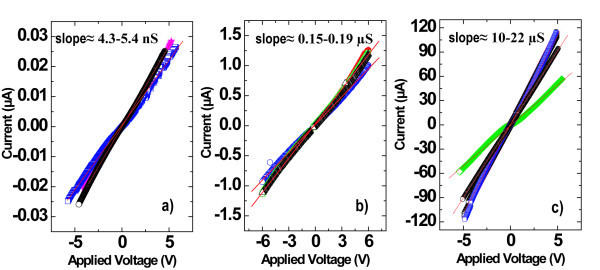
**CP-AFM *I*-*V *measurements on single phosphorus-doped SiNWs for different doping levels **: **(a) **undoped, **(b) **[*P*] ≈ 1 × 10^18 ^cm^-3^, and **(c) **[*P*] ≈ 1 × 10^20 ^cm^-3^

As illustrated in Figure 9, local *I*-*V *measurements were performed for each sample on top of the SiNW using a PtIr AFM tip. All the three samples show a linear behavior with inverse slopes of 1.9-2.3 × 10^8^, 5.3-6.7 × 10^6^, and 4.5-10 × 10^4 ^Ω, respectively, for the undoped, 1 × 10^18 ^and 1 × 10^20 ^for the doped samples. These values illustrate the total measured resistance *R*_tot _which can be decomposed as follows:

(1) $$R_{\text{tot}}\approx R_{\text{AFMtip}}+R_{\text{tip/SiNW}}+R_{\text{SiNW}}+R_{\text{back}},$$

where *R*_AFMtip _is the intrinsic resistance of the AFM tip, *R*_tip/SiNW _refers to the contact resistance involving the AFM tip and the SiNW, *R*_SiNW _designates the intrinsic resistance of the SiNW, and *R*_back _the back contact resistance between the highly doped silicon wafer and the SiNW. The intrinsic resistance of the SiNW (*R*_SiNW_) is given by *ρl*/*S *where *ρ*, *l*, and *S *are the resistivity, the length of the wire, and the wire sectional area, respectively.

The presence of contact resistance often implies the presence of a barrier which gives rise to diode-like behavior or sigmoidal *I*-*V *characteristics. In some cases, a linear dependence on applied bias can be measured indicating that the barrier resistance involved in the contact resistance can be neglected. The contact resistance only consists then in a geometrical resistance which depends on the electrical radius [23]. In order to estimate the geometrical resistance, the Wexler resistance model [24,25] was used, which describes the transition between the diffusive and ballistic transport regimes in constricted contacts.

Wexler formula is described as

(2) $$R_{\text{W}}=\frac{4\rho }{3\pi a}K+\frac{\rho }{2a}\Gamma (K),$$

where *K *= *λ*/*a *is the ratio of the carrier mean free path, *l*, to the electrical radius, *a*, and Γ(*K*) is a monotonous function that takes the value 1 at *K *= 0 and decreases slowly reaching the limit of 0.694.

For the estimation of *R*_tip/SiNW_, the electrical radius was chosen equal to 10 nm, and the electron mean free path in the range 1-80 nm assuming bulk silicon values. From these calculations, the resistivity values were estimated to be in the range of 20-40 Ω cm for the undoped sample, 0.1-1.2 Ω cm for the intermediate doped sample, and 0.008-0.016 Ω cm for the highly doped sample. In terms of electrically active phosphorus, it corresponds to 1-2 × 10^14^, 0.5-7 × 10^16^, and 2-6 × 10^18 ^cm^-3^, respectively. These values, extracted from bulk silicon values, indicate that the phosphorus incorporation is not fully activated despite the thermal anneal activation at 750°C. Recent results of CP-AFM show that phosphorus activation in SiNWs is enhanced at higher temperatures growth (*T *> 500°C) without the need of post-annealing treatment.

From the point of view of the CP-AFM measurements more accurate resistivity measurements could be achieved in the future making a pre-calibration of the technique using standard doped silicon wafers [26].

## Conclusion

In this study, CP-AFM was used to electrically characterize horizontal and vertical SiNWs. CP-AFM technique reveals itself as a powerful tool for sensing current inhomogeneities that were observed in horizontal SiNWs pointing out an internal microstructure. In addition, local *I*-*V *measurements allowed us to put in evidence a SCLC transport regime that could be assisted by traps.

The effect of phosphorus doping on the local contact resistance was evidenced for vertical SiNWs, and resistivity values were estimated indicating that phosphorus incorporation was not fully activated.

## Abbreviations

CP-AFM: conductive-probe atomic force microscopy; IPSLS: in-plane solid-liquid-solid; ITO: indium tin oxide; *I*-*V: *current-voltage; SCLC: space-charge limited current; SEM: scanning electron microscopy; SiNWs: silicon nanowires; VLS: vapor-liquid-solid.

## Competing interests

The authors declare that they have no competing interests.

## Authors' contributions

JA carried out CP-AFM measurements and drafted the manuscript. IN participated in the CP-AFM measurements for the horizontal SiNWs. MEGF and JPK participated in the guidance of the study and gived the corrections of manuscript. LY and PRIC grew the horizontal SiNWs and performed optical characterizations. SP, ER, CC, CM and JPS grew the vertical SiNWs, prepared them for the AFM analysis, and performed optical and electrical characterizations.
